# The Janus face of proliferating plasmablasts in dengue and COVID-19 infections

**DOI:** 10.3389/fimmu.2023.1068424

**Published:** 2023-08-11

**Authors:** Priya Nayak, Kavitha Mukund, Shankar Subramaniam

**Affiliations:** ^1^ Department of Bioengineering, University of California, San Diego, La Jolla, CA, United States; ^2^ Department of Cellular and Molecular Medicine, University of California, San Diego, La Jolla, CA, United States; ^3^ Department of Computer Science and Engineering, University of California, San Diego, La Jolla, CA, United States

**Keywords:** COVID-19, B-cells, dengue, plasmablasts, naive, memory, activation, inflammation

## Abstract

**Introduction:**

B cells play an integral role in the immune response to both dengue fever and COVID-19. Prior scRNAseq analyses of peripheral plasmablasts in COVID-19 have revealed a heterogeneous population with distinct cell subsets associated with proliferation; prior studies in patients with dengue fever have likewise shown the presence of proliferative pre-plasmablasts in the circulation. These findings may have implications for disease severity. In this study, we sought to gain a mechanistic understanding of the intracellular processes in naive and memory B cells that are associated with and may lead to an expanded proliferative plasmablast population in the circulation.

**Methods:**

We analyzed age-controlled (pediatric and adult), peripheral blood mononuclear cell scRNAseq datasets from patients infected with either dengue (primary or secondary) or COVID-19 (non-severe or severe) from previously published studies. Our preliminary analysis showed that pediatric patients with dengue and adults with COVID-19 had an expanded proliferative plasmablast (p-PB) population. By contrast, neither the adults with dengue nor the children with COVID-19 in our dataset had p-PBs. We used this distinctive preliminary signature to guide our analyses design and expanded our analyses to naive and memory B cells.

**Results:**

In age/disease conditions with and without p-PBs, we found differences in cell sensing and activation, including via the B cell receptor and downstream signal transduction. Likewise, inflammation was mediated differently: relative to groups without p-PBs, those with p-PBs had increased expression of interferon response and S100 genes (particularly severe COVID-19). Furthermore, several transcription factors at the nexus of activation, inflammation, and cell fate decisions were expressed differently in groups with and without p-PBs.

**Discussion:**

We used dengue and COVID-19 infections in adult and pediatric patients (focusing on naive B, memory B, and plasmablast cells) as a model to better understand the mechanisms that may give rise to p-PB populations in the circulation. Our results indicate that a more pro-inflammatory state in naive and memory B cells correlated with - and could influence the generation of- proliferating plasmablasts. Further exploration of these mechanisms will have implications for immune memory, vaccine development, and post-viral autoimmune syndromes.

## 1 Introduction

COVID-19 and dengue fever are viral illnesses with substantial global disease burden. COVID-19, caused by a novel coronavirus (SARS-CoV-2) with multiple emerging variants, has rapidly become pandemic (1). Dengue fever, a mosquito-borne illness, is endemic to tropical and subtropical regions, though the range is enlarging due to climatic changes. It is caused by a flavivirus with four serotypes (DENV1-4) (2). COVID-19 symptoms range from asymptomatic or mild in most cases, to severe requiring mechanical ventilation (1). Similarly, dengue fever symptoms range from asymptomatic or mild to severe hemorrhagic fever (2). However, while incidence of severe COVID-19 increases with age, children tend to have more severe dengue (3–5).

Although the viruses that cause COVID-19 and dengue fever have different target cells and routes of infection, dysfunction of B cell subsets, including naive B (NB), memory B (MB), and particularly of plasmablast (PB) cells, has been noted in both diseases (2, 6, 7). There is a substantial increase in the number of circulating plasmablasts during acute infection in both COVID-19 and dengue fever (6, 8, 9). Furthermore, an increase in plasmablast number is correlated with more severe disease. Prior scRNAseq analyses of peripheral plasmablasts in COVID-19 have revealed a heterogeneous population with distinct cell subsets associated with proliferation, robust response to interferon, and increased unfolded protein response to support antibody production (6, 10). In dengue fever, prior studies have shown a diverse repertoire of class-switched immunoglobulins, even with primary infection, as well as presence of proliferative pre-plasmablasts in the circulation (11).

While an increased number of plasmablasts following acute viral infection is expected, the degree to which this population is expanded in COVID-19 and dengue fever is notable. Likewise, the presence of a significant population of proliferative pre-plasmablasts in the circulation is notable and may have implications for disease severity. Increased presence of proliferating plasmablasts may serve as a metabolic sink and lead to lower energy availability during more severe disease (12). It is also hypothesized that increased plasmablast numbers may be linked to increased autoantibodies in COVID-19, with worsening outcomes (13, 14). Similarly, autoimmune syndromes caused by autoantibodies have been observed following dengue fever (15, 16). In addition to concern for antibody reactivity to autoantigens, there may also be cross-reactivity to other pathogens with implications for both mitigation and enhancement of disease. Although SARS-CoV-2 is a novel coronavirus, it has been hypothesized that there may be immune cross-reactivity between common circulating human coronaviruses and SARS-CoV-2 (17, 18). Children may derive more benefit from cross-reactivity since they are infected with circulating coronaviruses that cause the common cold more frequently than adults (19). However, it is unclear how long-lived the cross-reactivity may be, and whether it may result in cross-neutralization. The cross-reactivity may be mediated through both B and T cell subsets (20).

In dengue fever, by contrast, a secondary infection may paradoxically lead to more severe symptoms than primary infection due to antibody-dependent enhancement (ADE) (2), in which re-infection with a different DENV serotype leads to generation of non-neutralizing antibodies that promote cellular entry of opsonized viral particles via the fc gamma receptor; this may lead to enhanced inflammation and more severe symptoms in secondary dengue relative to primary dengue. SARS-CoV-2 has been observed to enter cells via fc gamma, but it is unclear whether this leads to increased COVID-19 severity (21, 22). DENV and SARS-CoV-2 co-infection have been reported in tropical and subtropical areas where dengue is endemic (23). There is emerging evidence for shared pathophysiology between the two viral illnesses: recent work indicates that patients previously infected with dengue had a lower mortality rate for subsequent COVID-19 (24, 25). Given the potential role of plasmablasts in the pathology of COVID-19 and dengue fever, a better understanding of the mechanisms that lead to expanded plasmablast populations, especially proliferative, could help in furthering an understanding of both diseases.

In this study, we subsequently sought to gain a mechanistic understanding of the intracellular processes in naive and memory B cells that are associated with, and may lead to an expanded proliferative plasmablast population in the circulation. In a preliminary analysis we observed a proliferative plasmablast (p-PB) population in children with dengue (particularly secondary) and in adults with COVID-19 (particularly severe); by contrast, there were relatively few p-PB in adults with dengue or children with COVID-19. We used this distinctive preliminary signature to guide our analyses design and expanded our analyses to naive and memory B cells which could explain the emergence of proliferative plasmablasts. We integrated publicly available peripheral B cell scRNAseq data from patients with primary dengue, secondary dengue, non-severe COVID-19, and severe COVID-19. We analyzed children and adults in parallel with age-matched controls, as both COVID-19 and dengue have age-dependent changes in symptoms (8, 9, 11, 26). We next compared gene expression patterns across age groups to delineate those that were correlated with the groups that had proliferative plasmablasts. We highlight gene networks in naive B, memory B, and plasmablast cells related to cell activation, inflammation, and transcription factors that mediate cross-talk between inflammatory cues and lineage determination to explain how naive and memory B cells may give rise to proliferative plasmablasts in the context of acute dengue and COVID-19 infections.

## 2 Results

### 2.1 scRNASeq data acquisition and analyses workflow

Single-cell RNA sequencing datasets of peripheral blood mononuclear cells from adults and children with COVID-19 and dengue were acquired from published studies (see Methods) (8, 9, 11, 26). Briefly, individual patients were selected based on their ages (5-15 years old for children or 20-50 for adults), time of sampling (within 10 days of symptom onset), and disease condition (primary and secondary dengue in children and adults, non-severe COVID-19 in children and adults, severe COVID-19 in adults only, and age-matched healthy controls for both). Patient meta data is shown in **Supplemental Figure 1**. Representative UMAP embeddings and corresponding expression of key immune cell markers are shown for one adult patient (**Supplemental Figures 2A, B**) and one pediatric patient (**Supplemental Figures 2C, D**).

**Figure 1:**
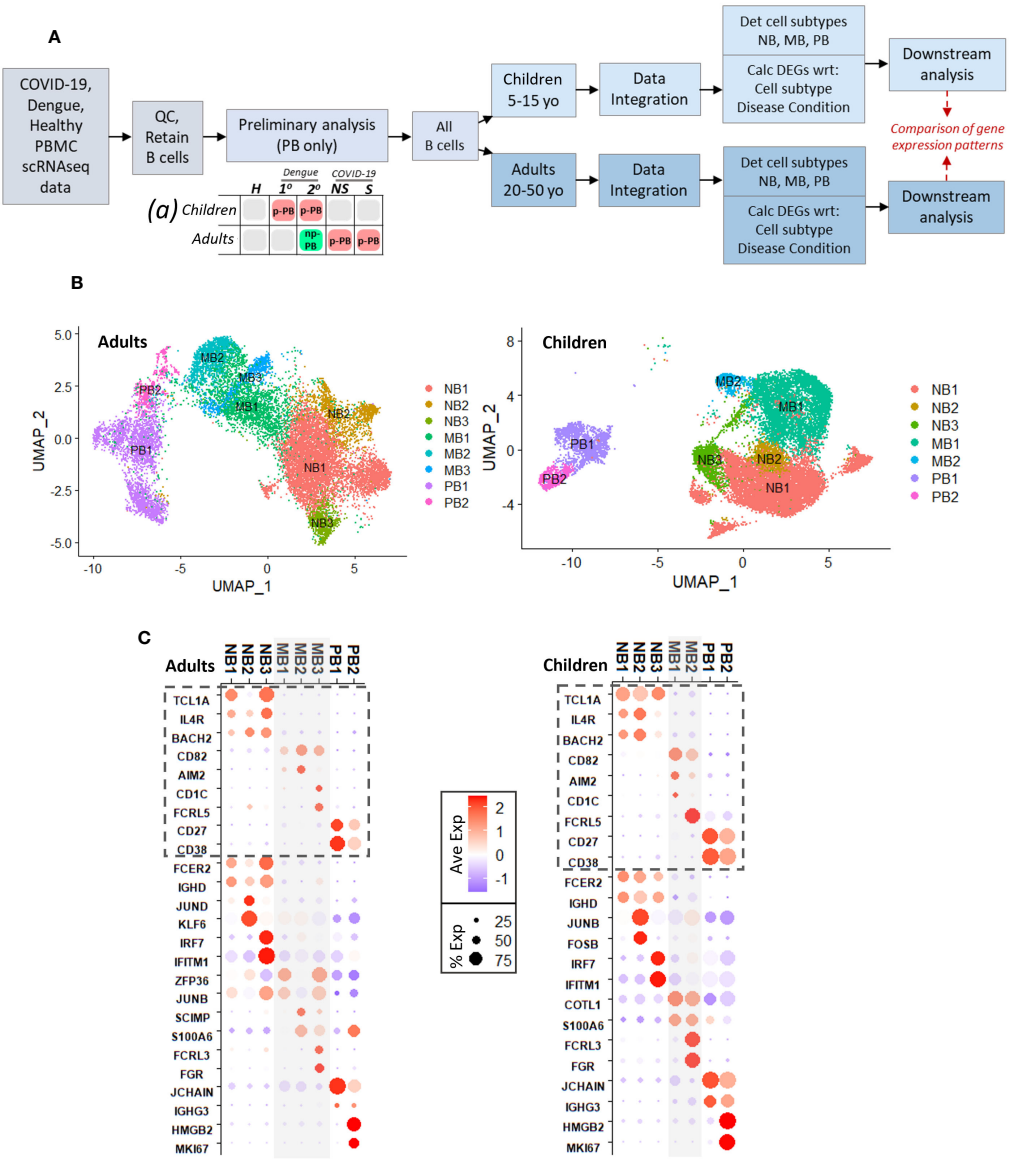
**(A)**. Schematic describing experimental design, with preliminary analysis and subsequent parallel analysis of pediatric and adult age groups, for disease conditions: primary dengue (1°), secondary dengue (2°), non-severe COVID-19 (NS), severe COVID-19 (S), age-matched healthy controls (H). Chart (a) describes results of preliminary analysis: red boxes represent presence of proliferative plasmablast (p-PB) population, green box represents non-proliferative (np-PB) population, grey boxes represent insufficient number of plasmablasts. **(B)**. UMAP embeddings for all B cells from adults (left) and children (right), showing cell subsets within naive B cells (NB), memory B cells (MB), and plasmablasts (PB). **(C)**. Dotplots showing key genes that delineate B cell types and subtypes for adults (left) and children (right). Genes with dashed box identify naive B, memory B, and plasmablast cells. Below dashed boxes, there is expression of selected cluster markers that distinguish each subtype within naive, memory, plasmablast cells, respectively. For example, in both children and adults NB3 was distinguished by expression on IFN-stimulated genes; PB2 was distinguished by expression of MKI67.

A schematic that describes the approach to analyses is shown in **Figure 1A**. A preliminary analysis including only plasmablasts showed two main plasmablast subsets in children and adults with dengue (primary and secondary) and COVID-19 (non-severe and severe) (**Supplemental Figures 2E, F**). The first subset (Prelim_PB1) was non-proliferative, with robust expression of immunoglobulin gene components. The second subset (Prelim_PB2) was proliferative, with MKI67 expression; however, within group Prelim_PB2, MKI67 was expressed only in adults with COVID-19 and children with dengue, but not in adults with dengue and children with COVID-19 (**Supplemental Figure 2G**, red box).

Based on the preliminary analysis, we broadened our approach to include all B cell subsets to better understand mechanisms that may lead to the emergence of proliferative plasmablasts. We separated the pediatric and adult age groups into separate, parallel analyses due to differences in symptoms in children and adults in both dengue and COVID-19, which may reflect age-related changes to the immune system. After data integration using the Seurat (27) package and determination of B cell subsets (naive, memory, plasmablasts), we calculated significant cluster markers (adjusted p-value <= 0.05) for each B cell subtype and, separately, for each disease condition within each age group (**Figure 1A**).

### 2.2 Comparable naive B, memory B, and plasmablast cell populations identified in adults and children, across dengue and COVID-19

Initial UMAP embeddings of B cells for adults and children are shown in **Supplemental Figures 3A, D**. For both adults and children, we considered only the clusters that were consistent with naive (NB) and memory B (MB) cells (CD19^+^, MS4A1/CD20^+^) or plasmablasts (PB) (CD27^+^, CD38^+^, CD19^-^) (**Supplemental Figures 3B, E**). Using similar expression patterns of top markers calculated for each numerical cluster (**Supplemental Figures 3C, F**), we grouped together clusters to determine the final designations for B cell subtypes in adults and children (**Figure 1B**). We selected significant markers (adj p <=0.05) that distinguished naive B, memory B, and plasmablast cells as described in previously published studies: Naive B cells were identified by expression of TCL1A, IL4R, and BACH2, while memory B cells expressed CD82, AIM2, CD1C, and FCRL5, and plasmablasts had CD27 and CD38 (**Figure 1C**, grey dashed boxes; top 15 markers for each cell subtype in **Supplementary Figures 4A, B**) (28–30).

Comparable cell subtypes (with respect to significant cluster markers, adj p <= 0.05) were identified in both age groups (**Figure 1C**; **Supplementary Figure 4**). Within naive B cells, three subtypes (NB1/2/3) were identified in adults and children. All the NB groups in adults and children had expression of FCER2 and IGHD, which are involved in environment sensing and immunoglobulin production, respectively (28). NB2 was distinguished by increased expression of AP-1 and related transcription factors (such as JUND, KLF6 in adults and JUNB, FOSB in children). AP-1 transcription factors play a role in a variety of processes in B cells including activation, inflammation, and differentiation (31, 32). Meanwhile NB3 was notable for increased expression of several interferon-stimulated genes, including IFITM1 and IRF7 in both children and adults. Within memory cells, adults had three subtypes (MB1/2/3) while children had two (MB1/2); however, across the age groups, there were similarities in the cluster markers (adj. p <= 0.05) that differentiated the memory B cell subtypes (**Supplementary Figure 4**; **Figure 1C**). Subtypes MB2/3 in adults and MB1/2 in children had expression of S100 genes (such as S100A4, S100A6, S100A10) that function as intra- and extra-cellular calcium binding proteins that play a role in a wide range of immune cell processes (33, 34). In addition, genes FCRL3 and FGR were cluster markers that distinguished MB3 in adults and MB2 in children and play a role in B cell activation and inflammation, respectively (35, 36). Finally, in both adults and children, there were two plasmablast subtypes (PB1/2): PB1 had robust expression of immunoglobulin components (such as JCHAIN, IGHG3), while PB2 had expression of genes relating to proliferation, including MKI67. The presence of non-proliferative and proliferative PB populations was consistent with the preliminary analysis.

### 2.3 Increased expression of interferon-stimulated, variable Ig chain components, and S100 genes differentiated primary dengue/non-severe COVID-19, secondary dengue, and severe COVID-19, respectively

**Figure 2:**
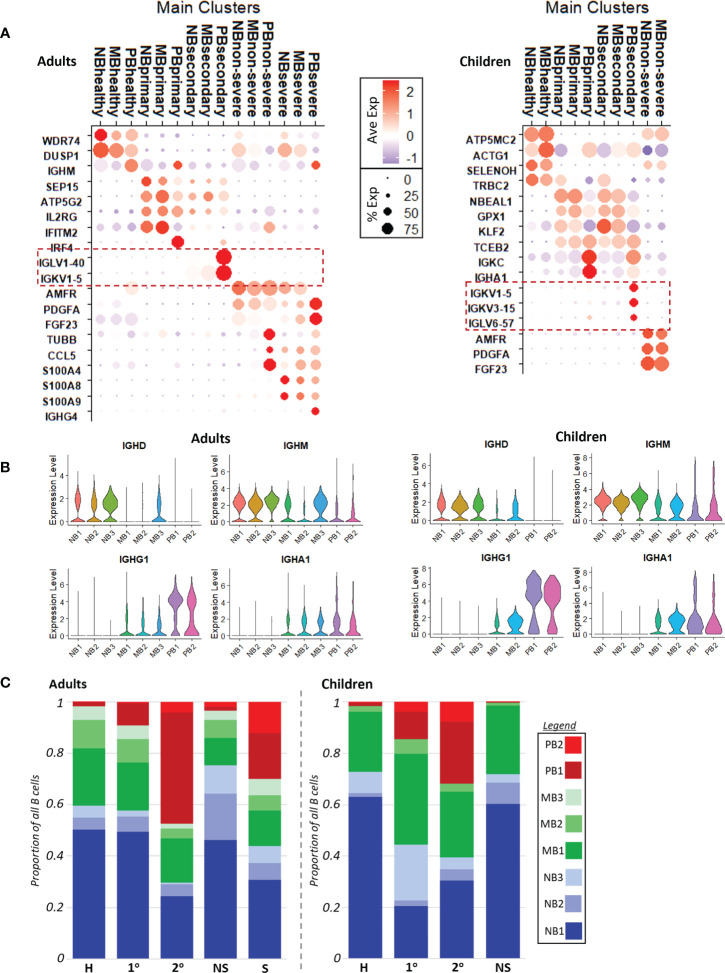
**(A)**. Dotplots with key cluster markers calculated with respect to disease conditions. Red dashed boxes highlight variable Ig chain expression only in secondary dengue, in both adults (left) and children (right). NB, naive B cells; MB, memory B cells; PB, plasmablasts; severe, severe COVID-19; non-severe, non-severe COVID-19; secondary, secondary dengue; primary, primary dengue; healthy, healthy age-matched control. **(B)**. Volcano plot showing the expression of immunoglobulin heavy chains IGHD, IGHM, IGHG1, IGHA1 with respect to B cell subtype in adults (left) and children (right). **(C)**. Cell counts show relative cell abundances with respect to B cell subtype and disease condition in adults (left) and children (right).

To better identify genes that differentiated disease conditions, we calculated markers (p adj <= 0.05) with respect to disease condition, across B cell subtypes. The top five markers for each disease condition, within each of naive B, memory B, and plasmablast cells for adults and children separately, are shown in **Supplemental Figure 5**. In both children and adults, primary and secondary dengue had more similar gene expression, while non-severe and severe COVID-19 were more similar. Selected genes that reflected the changing faces of inflammation across disease conditions, as consistent with the literature and gene ontology analysis, are shown in **Figure 2A** (with gene ontology terms in **Supplemental Figures 6A, B**).

In children and adults, gene ontology biological process (GOBP) terms calculated (p adj <= 0.05) using markers reflected increased interferon signaling in primary dengue, driven by increased expression of genes including IFITM2 and IRF4 in adults and GPX1 and TCEB2 in children. In secondary dengue in children and adults, there was significant increased expression of variable Ig chain components (**Figure 2A**, dotted red boxes); corresponding GOBP terms related to protein translation to support antibody production were also enriched (**Supplementary Figures 6A, B**), likely alluding to the reactivation of immune memory and expansion of cells that were selected in a prior infection; neither primary dengue nor COVID-19 had expression of the same variable chain component in a substantial proportion of cells, which was more suggestive of initial infection than immune memory reactivation (37).

Non-severe COVID-19 was distinguished from other disease conditions in both children and adults by expression of PDGFA, AMFR, and FGF23, markers that have been observed to be upregulated in prior studies of acute COVID-19 infection (**Figure 2A**) (38, 39). In GOBP analysis, adults with non-severe COVID-19 had enrichment of terms related to interferon signaling, while children with non-severe COVID-19 did not (**Supplementary Figures 6A, B**). This may reflect the milder course of COVID-19 in children relative to adults. Finally, severe COVID-19 (only in adults) was distinguished by expression of S100 genes, such as S100A4/8/9, and had a more robust inflammatory response as reflected in GOBP enrichment terms (**Supplemental Figure 6A**), consistent with earlier reports (6). In contrast to secondary dengue, neither COVID-19 group showed increased expression of variable Ig chain components; this could imply either a potential lack of immune cross-reactivity to SARS-CoV-2 (as could have been elicited due to prior infection with another coronavirus), or that immune memory to prior coronavirus infection may be too short-lived to be readily captured in these datasets (17–19).

Invariable immunoglobulin heavy chain expression progressed as expected across naive B cells with IGHD/IGHM expression, to increasing expression of IGHG/IGHA in memory B cells, to predominantly IGHG/IGHA expression in plasmablasts, as class-switching occurs (**Figure 2B**) (40).

### 2.4 Increased proliferative plasmablasts in adults with severe COVID-19 and children with secondary dengue

We compared the relative proportions of each B cell subtype with respect to disease condition within adults and children (**Figure 2C**). In our dataset, adult patients with secondary dengue and severe COVID-19 had the most markedly different B cell abundance profiles relative to healthy, primary dengue, and non-severe COVID-19. Secondary dengue in adults had a relative increase of non-proliferative plasmablasts (PB1); taken together with increased expression of variable Ig chains in secondary dengue (**Figure 2A**), this likely reflects, at least in part, re-activation of immune memory from a prior dengue infection and expansion of clonal cells (37), though not all expansion may be attributable to immune reactivation (4). In adults with severe COVID-19, there was also an expansion of non-proliferative PBs (PB1), along with a substantial relative increase in the number of proliferating plasmablasts (PB2). In both adults with secondary dengue and severe COVID-19, there was a corresponding decrease in the proportion of naive B cells. By contrast, children with primary and secondary dengue had cell abundance profiles most different from their corresponding healthy controls. As in adults, there was expansion of non-proliferative plasmablasts (PB1) in dengue, as well as an increase in proliferative plasmablasts (PB2) in children with dengue (secondary more than primary). This indicates the presence of a proliferative plasmablast population in adults with severe COVID-19 and children with dengue (secondary more than primary).

### 2.5 Functional relationships between B cell activation, inflammation, and transcriptional regulation

We next sought to better understand the transcriptional and functional networks in naïve, memory, and plasmablast cells that are correlated with and may drive emergence of proliferating plasmablasts (p-PB). We first identified transcription factors as defined by Lambert et al. (41) within significant cluster markers (p adj <= 0.05) calculated with respect to cell subtype (of which the top 15 for each group are shown in **Supplementary Figure 4**). For each of naive B, memory B, and plasmablast cells, we initially considered transcription factors present in both children and adults (**Supplemental Figure 6C**), as well as their targets (as curated in Transfac) that were also within significant cluster markers. We applied unsupervised K-means clustering to group genes with respect to their expression in each age and disease condition (**Supplemental Figure 7**). Two resulting clusters captured the overarching pattern of expression: one group of genes had increased expression in groups with p-PBs and one groups of genes had increased expression in groups without p-PBs. We mapped functional relationships between TFs and their targets to better understand how variations in key areas of cell activation and inflammation could influence the emergence of p-PBs (see methods for more details). In the following sections, we describe networks for naive B, memory B, and plasmablast cells (with extended networks in **Supplemental Figures 8–10**).

#### 2.5.1 Naive B cells

**Figure 3:**
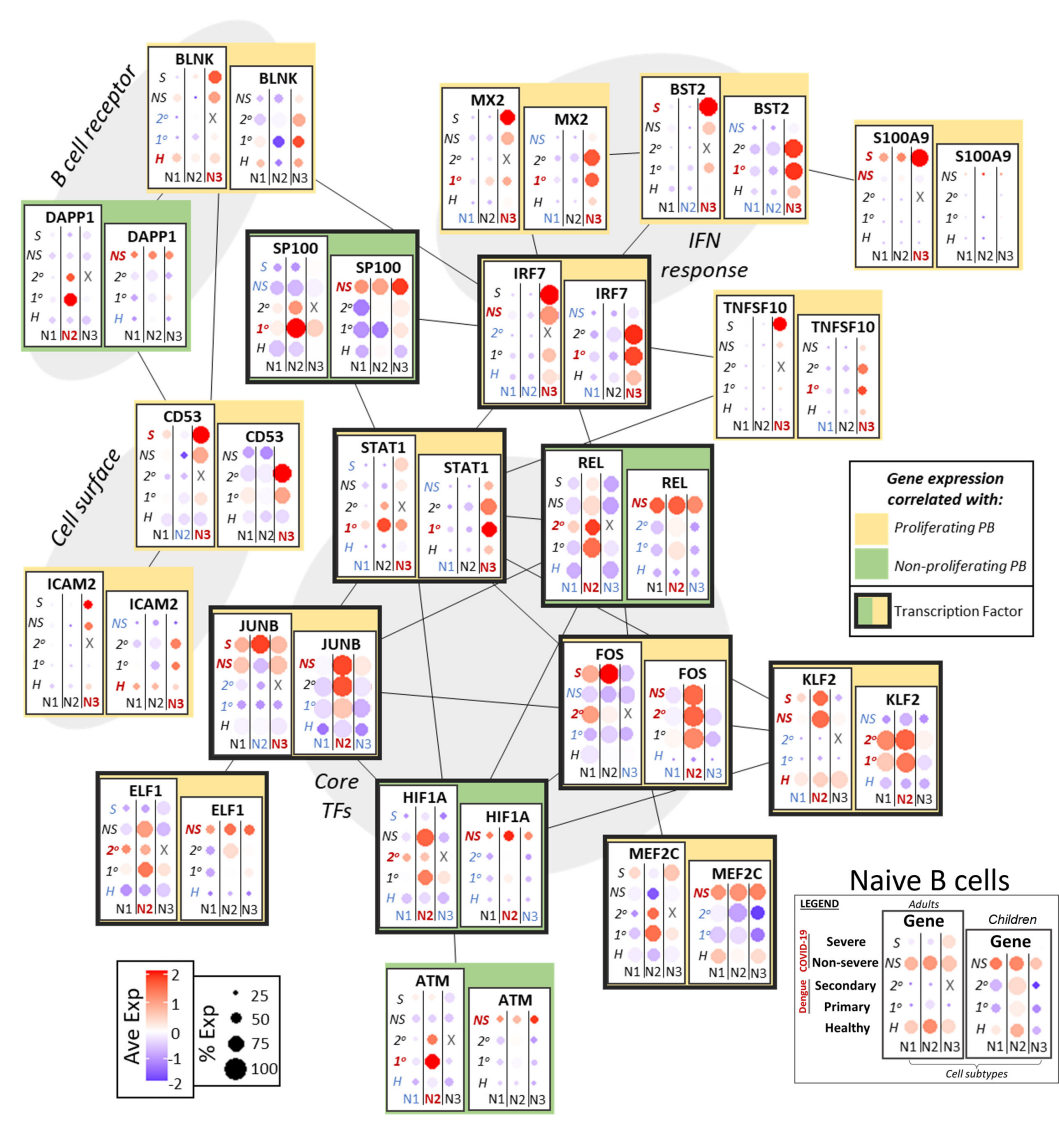
Network of functional associations between key genes in naive B cell function and differentiation, including core transcription factors (TFs), interferon (IFN) response, B cell receptor components, and cell surface molecules. Within each box, dotplots represent gene expression in adults (left) and children (right), with respect to disease conditions: primary dengue (1°), secondary dengue (2°), non-severe COVID-19 (NS), severe COVID-19 (S), age-matched healthy controls (H). N1, N2, N3 are the three subsets of naive B cells as defined previously. Red and blue highlighting of disease conditions and cell subtypes within each box represents whether the gene was significantly up or down regulated, respectively, in that group. The yellow or green color represents whether expression of the gene across ages and disease conditions correlated with emergence of a proliferating or non-proliferating plasmablast population. Finally, connections between genes represent functional relationships as determined using STRINGdb.

Naive B cells that are activated during the acute phase of an infection can give rise to both memory B cells and plasmablasts (**Figure 3**) (42). B cell receptor (BCR) component BLNK, tetraspanin CD53, and adhesion molecule ICAM2 had expression that correlated with p-PBs, while BCR component DAPP1 correlated with non-proliferating PBs (np-PBs). Interferon response genes, including MX2, BST2, and IRF7, were notably upregulated in the NB3 subpopulation and correlated with p-PBs in both adults and children. Though they both modulate inflammation, unlike IRF7, SP100 expression was correlated with np-PB populations. S100A9 was upregulated only in adults with severe COVID-19, while upregulation of TNFSF10 (TRAIL) was correlated with p-PB, indicating more robust inflammation in these groups. Several TFs that bridge inflammatory processes (AP-1 TFs, such as JUNB and FOS, and KLF2) and lineage determination (MEF2C, ELF1) had increased expression that correlated with p-PB groups. STAT1 had mixed expression: its expression was better correlated with p-PB in children than in adults. By contrast, genes that modulate (REL in NF-kB) and mitigate (HIF1A, ATM) stress had expression that correlated with np-PB populations. Taken together, this may indicate that sensing of inflammation and activation in naive B cells, as well as downstream TFs that influence lineage determination, correlate with the presence of and may play a role in the generation of a p-PB population.

#### 2.5.2 Memory B cells

**Figure 4:**
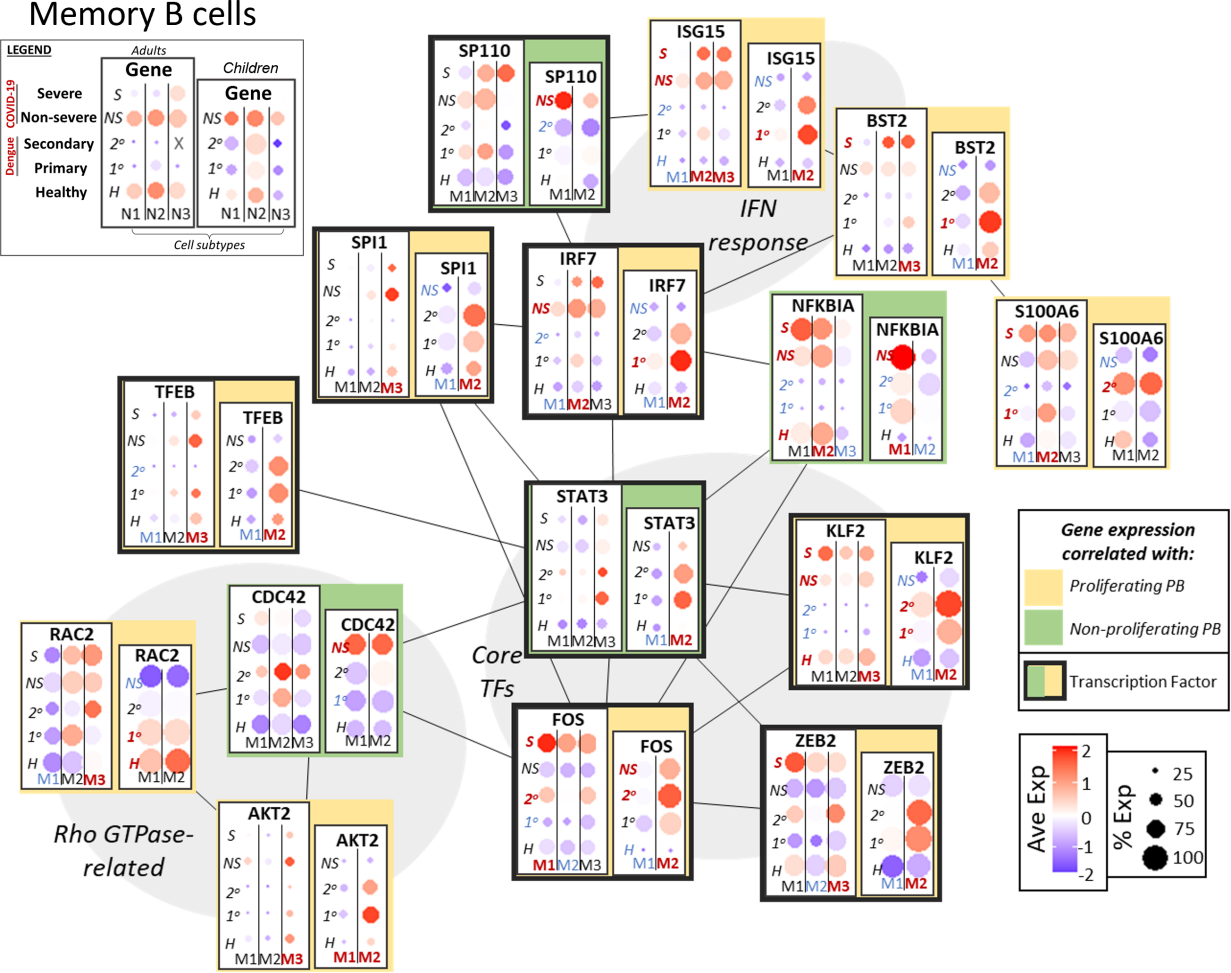
Network of functional associations between key genes in memory B cell function and differentiation, including core transcription factors (TFs), interferon (IFN) response, and Rho GTPase-related. Within each box, dotplots represent gene expression in adults (left) and children (right), with respect to disease conditions: primary dengue (1°), secondary dengue (2°), non-severe COVID-19 (NS), severe COVID-19 (S), age-matched healthy controls (H). M1, M2, M3 are the subsets of memory B cells as defined previously (M1-3 in adults, M1/2 in children). Red and blue highlighting of disease conditions and cell subtypes within each box represents whether the gene was significantly up or down regulated, respectively, in that group. The yellow or green color represents whether expression of the gene across ages and disease conditions correlated with emergence of a proliferating or non-proliferating plasmablast population. Finally, connections between genes represent functional relationships as determined using STRINGdb.

In acute infection, memory B cells arise from activated naive B cells and re-activation of resting memory B cells; memory B cells in turn can give rise to plasmablasts (42). As noted above, expression of multiple variable chain immunoglobulin components may be evidence of immunological memory in the case of secondary dengue infection in adults and children. We investigated the transcriptional network and targets related to interferon response and cell activation/differentiation to correlate expression of key genes to populations with p-PBs (**Figure 4**).

Interferon response genes, including ISG15 and BST2, had increased expression in the same groups that also had p-PBs (adults with COVID-19, children with dengue). Expression of S100A6 was correlated with p-PB groups, while NFKBIA had mixed expression. Rho GTPase CDC42, which plays a role in B cell development (43), had expression correlated with np-PBs; meanwhile RAC2 and AKT2 (a kinase that could modulate Rho GTPase activity) expression was correlated with p-PBs, indicating a possible role for Rho GTPases in the context of PB generation in acute infection. Furthermore, core TFs that mediate crosstalk between inflammation and cell differentiation had expression that was correlated with groups with p-PB (adults with COVID-19, children with dengue): FOS and KLF2 had increased expression in groups with p-PBs, as did the TFs ZEB2, which plays a role in B cell maturity and EMT, and TFEB, involved in autophagy (44). IRF1 and SPI1 (PU.1) expression correlated with p-PBs, while SP110 expression correlated with np-PBs. STAT3 had mixed expression, as it was correlated with p-PBs in children but not in adults. Similar to what we observed in naive B cells, this could indicate that variation in inflammation sensing, signal transduction, and transcriptional regulation may play a role in subsequent generation of a p-PB population in the peripheral circulation.

#### 2.5.3 Plasmablasts

**Figure 5:**
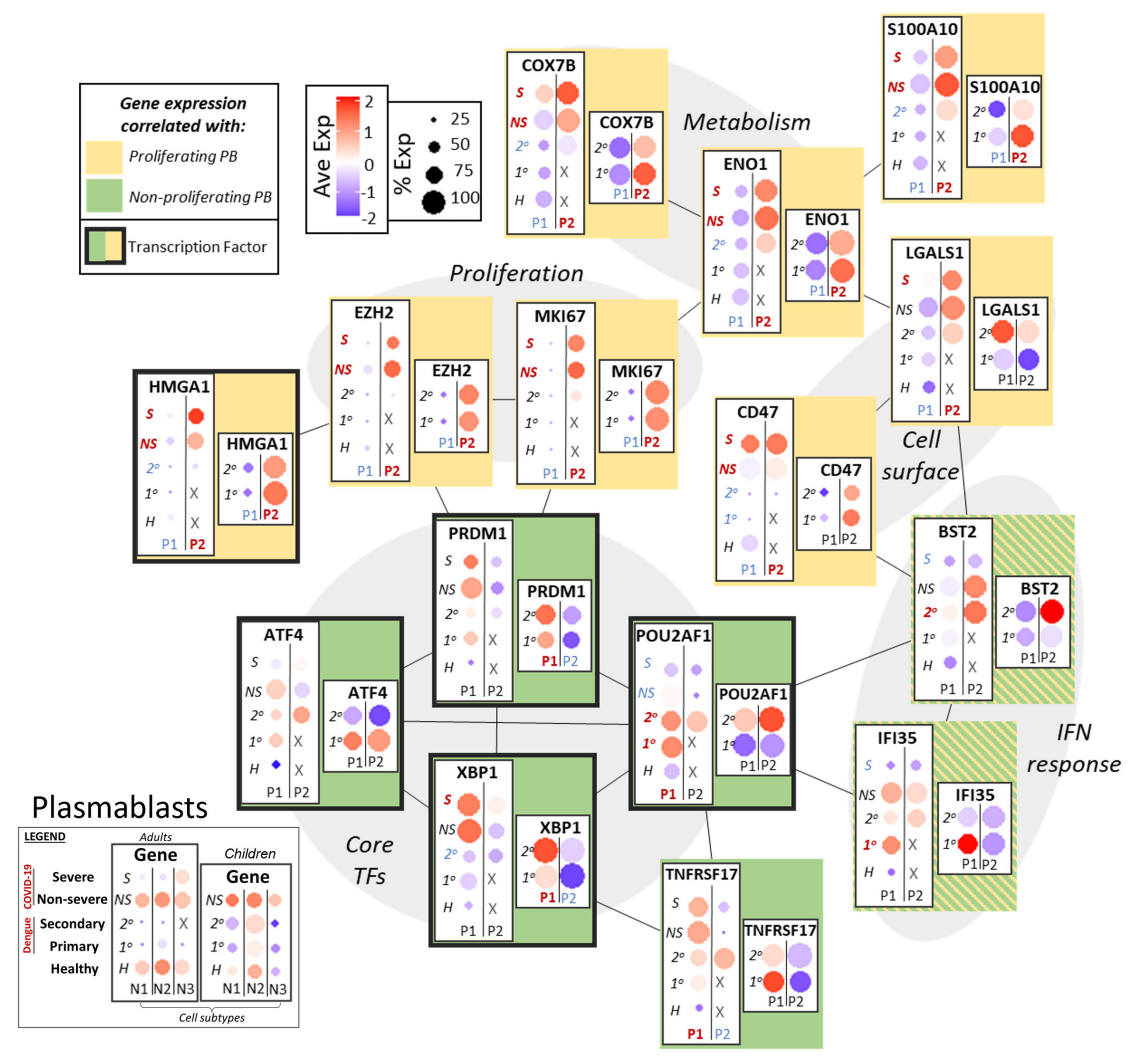
Network of functional associations between key genes in plasmablast function and differentiation, including core transcription factors (TFs), interferon (IFN) response, proliferation, metabolism and cell surface molecules. Within each box, dotplots represent gene expression in adults (left) and children (right), with respect to disease conditions: primary dengue (1°), secondary dengue (2°), non-severe COVID-19 (NS), severe COVID-19 (S), age-matched healthy controls (H). P1, P2 are the two subsets of plasmablasts as defined previously. Red and blue highlighting of disease conditions and cell subtypes within each box represents whether the gene was significantly up or down regulated, respectively, in that group. The yellow or green color represents whether expression of the gene across ages and disease conditions correlated with gene signatures of proliferating or non-proliferating plasmablasts. Finally, connections between genes represent functional relationships as determined using STRINGdb.

As noted above, expression of EZH2 and MKI67 in adults with COVID-19 (particularly severe) and children with dengue (particularly secondary) indicated the presence of proliferative, “pre-plasmablast” cells in the peripheral circulation of these groups (**Figure 5**) (45). Expression of epigenetic regulators and metabolic factors that support proliferation had increased expression in groups with p-PB: Non-histone chromatin factor HMGA1, which promotes proliferation, was upregulated in the p-PB groups, as were genes that play a role in oxidative phosphorylation (COX7B) and glycolytic metabolism (ENO1). LGALS1 and CD47, a surface galectin and integrin respectively, that may promote early plasmablast commitment, were likewise upregulated in the p-PB groups (8, 46). As in naive and memory B cells, S100 genes (such as S100A10) were more upregulated in in the p-PB groups, especially severe COVID-19. By contrast to naive and memory B cells that had expression of IFN response genes in the same groups that had p-PB, IFN response genes had variable expression in plasmablasts. BST2 was expressed more in the p-PB group, while IFI35 had increased expression in the np-PB, with variable disease-dependence.

B cell maturation antigen TNFRSF17 (BCMA) had increased expression in the np-PB groups, (COVID-19 in adults and dengue in children) and may reflect the increased maturity of the np-PB groups. Likewise, core transcription factors that promote commitment to the plasmablast lineage, including PRDM1 (BLIMP-1) and XBP1 had increased expression in the np-PB group relative to p-PB. ATF4, which regulates the unfolded protein response to support increased antibody production, and POU2AF1, which promotes plasmablast maturation, also had increased expression in the np-PB groups. In addition to highlighting possible disease-dependent differences in plasmablast generation and maturation, this may also indicate the relative immaturity of the p-PB population compared to np-PB.

### 2.6 Independent dengue dataset has similar gene expression profiles in groups with and without p-PBs

**Figure 6:**
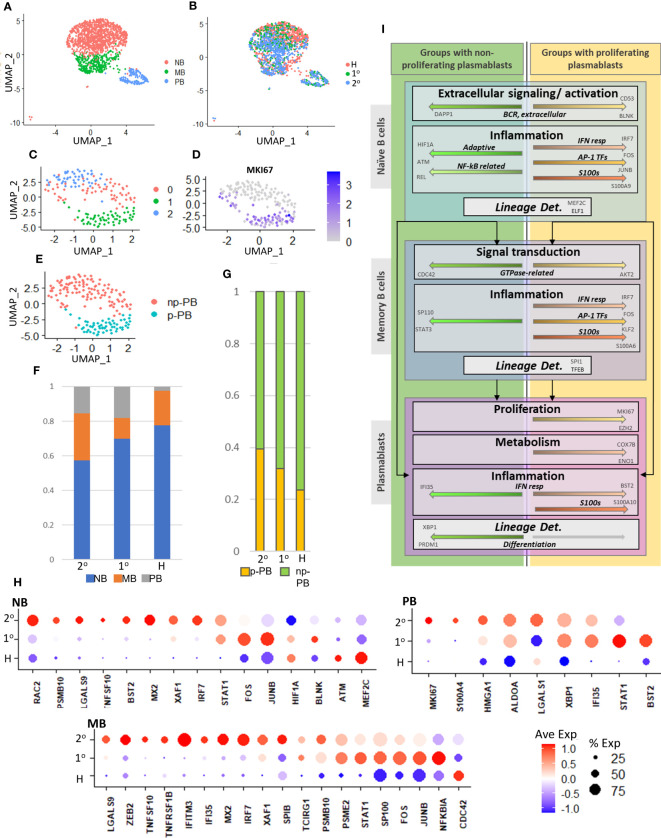
**(A)**. UMAP representation of naive B (NB), memory B (MB), and plasmablast (PB) cells from an independent dataset of adult patients with dengue (47). **(B)**. UMAP representation shows distribution of cells from healthy (H), primary (1°), and secondary (2°) dengue patients. **(C)**. UMAP representation of subset and reclustered plasmablasts from part **(A)**. **(D)**. Expression of MKI67 in reclustered plasmablasts. **(E)**. UMAP representation of reclustered plasmablasts. MKI67^+^ cluster from part **(D)** is designated as proliferating (p-PB), while MKI67^-^ is designated non-proliferating (np-PB). **(F)**. Relative cell abundances with respect to B cell subtype (NB, MB, PB) for healthy (H), primary (1°), and secondary (2°) dengue patients. **(G)**. Relative abundances of proliferating (p-PB) and non-proliferating (np-PB) plasmablasts for healthy (H), primary (1°), and secondary (2°) dengue patients. **(H)**. Expression of key genes highlighted in **Figures 3**–**5** in independent data set, in B cell subsets (NB, MB, PB) with respect to disease condition (healthy (H), primary (1°), and secondary (2°) dengue). **(I)**. Chart to summarize key intracellular mechanisms (within naive B, memory B, and plasmablast cells) that may influence lineage determination and give rise to circulatory proliferating plasmablasts in adults with COVID-19 and children with dengue. Top box represents naive B cells, middle box represents memory B, bottom box represents plasmablasts.

To further bolster the association between increased presence of p-PBs and the gene signatures described above, we analyzed an additional independent dataset consisting of scRNA seq of peripheral blood mononuclear cells from patients with dengue, published in Zanini et al. (47). Patient metadata is summarized in **Supplementary Figure 11A**. Briefly, four adult patients with dengue (two with primary dengue, two with secondary dengue) 2-5 days from onset of symptoms and four age-matched healthy controls were included. Initial scRNA seq data quality control and integration were performed with Seurat, as described in methods, and B cell subsets were selected based on expression of CD19, MS4A1 for naive and memory B cells and CD27, CD38 for plasmablasts (**Supplementary Figures 11B, C**). We reclustered the B cell subsets and noted a proliferative MKI67^+^ cluster (**Supplementary Figures 11D, E**). We retained clusters consistent with B cells based on significant cluster markers (p <= 0.05) (**Supplementary Figure 11F**) and determined naive B, memory B, and plasmablast clusters with healthy, primary dengue, and secondary dengue cells distributed throughout all clusters (**Figures 6A, B**). To better delineate proliferating plasmablasts, we further reclustered the plasmablast cells and designated the MKI67^+^ cluster p-PB (**Figures 6C–E**). We noted a relative decrease in NB cells and a corresponding increase in MB and PB cells in primary and secondary dengue relative to healthy controls (**Figure 6F**). While the proportion of B cells that are PB in primary and secondary dengue groups is similar, the proportion of plasmablasts that are p-PB are increased in secondary dengue, relative to both primary dengue and healthy control groups (**Figure 6G**). In this independent dataset, secondary dengue represented the group with increased p-PBs, while healthy had less p-PBs and primary dengue was intermediate. We then checked whether key genes identified in networks in **Figures 3**–**5** had similar expression patterns in groups that did and did not have p-PBs. In naive B, memory B and plasmablast, multiple key genes had expression patterns consistent with what we observed in the initial dataset (**Figure 6H**), particularly genes related to inflammation, such as MX2, IRF7, and transcription factors that mediate inflammation and may play a role in differentiation, including FOS, STAT1, XBP1, and SP100.

## 3 Discussion

B cell activation is a crucial part of the immune response in both acute dengue fever and COVID-19 (6, 8). We observed a population of proliferative plasmablasts (p-PBs) in the peripheral blood of adults with COVID-19 (particularly severe), and children with dengue (particularly secondary), but not in the other disease conditions (adults with dengue, children with COVID-19). We therefore used dengue and COVID-19 infections in adult and pediatric patients (focusing on naive B, memory B, and plasmablast cells) as a model to better understand the mechanisms that may give rise to p-PB populations in the circulation.

We investigated gene expression patterns related to B cell activation, inflammation, signal transduction, and transcriptional regulation that could modulate cell fate decisions and may give rise to p-PBs (**Figures 3**–**5**). This is synthesized in **Figure 6B**: Differences in expression of B cell receptor components and Rho GTPases in naive and memory B cells, respectively, indicate that variations in extracellular sensing and signal transduction could influence preferential differentiation to circulating p-PBs. Downstream response to inflammatory cues was likewise distinct in groups that did and did not have p-PBs. Pro-inflammatory S100 family and interferon-stimulated genes were upregulated in naive and memory B cells in the groups that also had p-PBs. By contrast, genes that modulate- and may mitigate- inflammation (such as ATM) were more upregulated in groups that did not have p-PBs. Crucially, the expression of transcription factors that mediate crosstalk between pro- and anti-inflammatory processes, proliferation, and lineage determination varied in groups that do and do not have p-PBs, including several AP-1 TFs (such as FOS and JUNB) that were upregulated in groups that had p-PBs. Altogether, our results indicate that a more pro-inflammatory state in naive and memory B cells might shift differentiation towards proliferating plasmablasts in our model system of pediatric and adult cases of dengue and COVID-19.

Data selection presents a challenge for this type of study. We leveraged COVID-19 and dengue patients as model systems to better understand gene expression changes in B cell subsets that correlate with, and may lead to, emergence of p-PB populations in the circulation. However, this does not necessarily imply the cell and gene signatures are truly a signature of the particular disease state. Several factors can make this determination difficult including small numbers of patients, limited clinical data to better track disease trajectory (including longer term follow up), and patient-to-patient variability. In our study, we therefore correlate the increased presence of a cell population (proliferating plasmablasts) in the circulation with gene expression signatures in B cell subsets that may explain their emergence. In addition, we showed similar gene expression patterns in an independent dataset of adult patients with dengue (**Figures 6A–H**). These analyses provide a starting point for future studies to further elucidate disease-specific signatures. Furthermore, we have made efforts to mitigate variation by controlling for age, disease, and sampling time (separately in adults and children), and by use of Seurat’s data integration features.

Use of peripheral blood cells presents a further challenge as most lymphocyte activation, maturation, and selection takes place within secondary lymphoid organs. Therefore, a peripheral blood sample of B cells represents a mixture of cells that are re-circulating through the lymphatic and circulatory system, in various stages of activation, inflammation, homing, and maturity. In dengue, there is evidence of direct infection of dendritic and monocytic cells (2), and in COVID-19 there is likewise evidence of dendritic cell, and monocyte dysfunction (6) as well as histological evidence of blood vessel dysfunction within lymph nodes (48) A hypothetical mechanism of action that takes into account B cell generation within lymph nodes, and that may be promoted by the crosstalk between transcription factors that determine cell fate and pro-inflammatory genes we observed, could serve as a basis for further questions. Future studies of peripheral B cells could focus on time points early in the course of symptoms to better capture cell specification at the outset of illness. Additional studies could focus on the lymph node as a whole and consider the multiple cell types involved in B lymphocyte activation, maturation, and selection, as this has implications for development of immunological memory, autoimmunity, and vaccine development (10, 49).

## 4 Materials and methods

### 4.1 Single-cell RNAseq data acquisition

Single-cell RNA seq datasets were acquired from publicly available repositories. Data for adults with COVID-19, children with COVID-19, and adult controls was acquired from Ren et al. (9) with GEO accession number GSE158055. Data from adults with dengue was acquired from Rouers et al. (8) with GEO accession number GSE172180. Data from children with dengue was acquired from Waickman et al. (11) with GEO accession number GSE145307. Healthy pediatric controls were acquired from Wang et al. (26) with GEO accession number GSE168732. An additional independent dataset to corroborate our findings was obtained from Zanini et al. (47) with GEO accession number GSE116672 (See **Supplemental Figure 11A** for patient characteristics). All datasets were derived from peripheral blood mononuclear cells.

### 4.2 Patient characteristics

Individual patients and controls were selected based on age-matching, disease-matching, and time to sampling post-infection. Pediatric patient ages ranged from 5-15 years old. Adult patient ages ranged from 20-50 years old. Disease categories for pediatric patients were primary dengue, secondary dengue, non-severe COVID-19, and healthy controls, while disease categories for adults were primary dengue, secondary dengue, non-severe COVID-19, severe COVID-19, and healthy controls; there were no children with severe COVID-19 in our dataset. Disease category designations were adopted from their respective studies. Time to sampling for both adults and children was up to 10 days after symptom onset.

### 4.3 scRNASeq data quality control

Seurat v3 was used for analysis of scRNA seq data (27). Individual Seurat objects were created for each patient to better assess cell number and cell subtype clusters on a per-patient basis. Cell subtypes were assessed using lineage identifier genes (**Supplemental Figure 2**). Data filtering at the cell and gene level was performed. Cells with more than 25% mitochondrial transcripts, less than 500 transcripts, and log10genes/UMI less than 80% were excluded from analysis. Genes that were expressed in less than 10 cells were excluded. B cell clusters were selected from each patient using B cell markers (CD19+/CD20 (MS4A1)+ for naive and memory B cells; CD27+/CD38+ for plasmablasts). Data from adult and pediatric patients were subsequently processed and analyzed in parallel to better control for age-related immune system changes.

### 4.4 scRNASeq data integration, cluster marker identification, and visualization

Prior to integration, Seurat objects for each patient from the same disease condition were merged; since each disease condition corresponded to a unique study, this also served to divide the pre-integration Seurat objects by study of origin. Subsequent integration anchor calculation therefore accounted for both disease condition and study of origin. Integration anchors were computed using Seurat’s FindIntegrationAnchors function, followed by creation of a Seurat object using the IntegrateData function. The integrated Seurat objects (one for adults, one for children) were then scaled, dimensions reduced, and cell clusters were determined using the standard Seurat workflow. We calculated significant genes that defined each cell subtype category (i.e., NB1/2/3, MB1/2/3, PB1/2) and each disease condition (e.g., primary, secondary dengue; non-severe, severe COVID-19; healthy) separately. We used Seurat’s FindAllMarkers function with significant genes at adjusted p-value <=0.05. UMAP representations, dotplot, and violin plots were generated using Seurat’s DimPlot, DotPlot, and VlnPlot functions, respectively. To assess whether genes were expressed more in groups with or without p-PBs, k means clustering was implemented in R using the “kmeans”function with average gene expression values with respect to disease condition as input. Resulting clusters were visualized with the “factoextra” package.

### 4.5 Gene ontology enrichment analysis

Gene set enrichment for significant cluster markers (p-adj <= 0.05) that defined disease conditions was performed using gene ontology biologic process (GOBP) enrichment, as implemented by the Enrichr tool (50). Significant enrichment terms had p-adj <= 0.05.

### 4.6 Functional interaction networks

Transcription factors were identified from significant cluster markers (p-adj <= 0.05) using a list of curated TFs as defined in Lambert et al. (41). TFs that were present in both children and adults for each cell type (naive B, memory B, plasmablast cells) were retained. Targets of selected TFs were identified from the list of significant genes using TF target lists from the TRANSFAC curated transcription factor dataset (51). Functional connections were established jointly with STRINGdb and information from the literature (52).

## Data availability statement

The original contributions presented in the study are included in the article/**Supplementary Material**. Further inquiries can be directed to the corresponding author.

## Author contributions

Conceptualization: PN, SS. Methodology: PN, SS. Investigation: PN, KM. Funding acquisition: SS. Supervision: SS. Writing – original draft: PN. Writing – review and editing: PN, KM, SS. All authors contributed to the article and approved the submitted version.
