# Supplementary material for: The Janus face of proliferating plasmablasts in dengue and COVID-19 infections
Source: Front Immunol. 2023 Aug 11;14:1068424. doi: 10.3389/fimmu.2023.1068424 (PMC10450630; doi:10.3389/fimmu.2023.1068424)
Supplement: Supplementary file 1 [file DataSheet_1.docx]

Supplementary Material


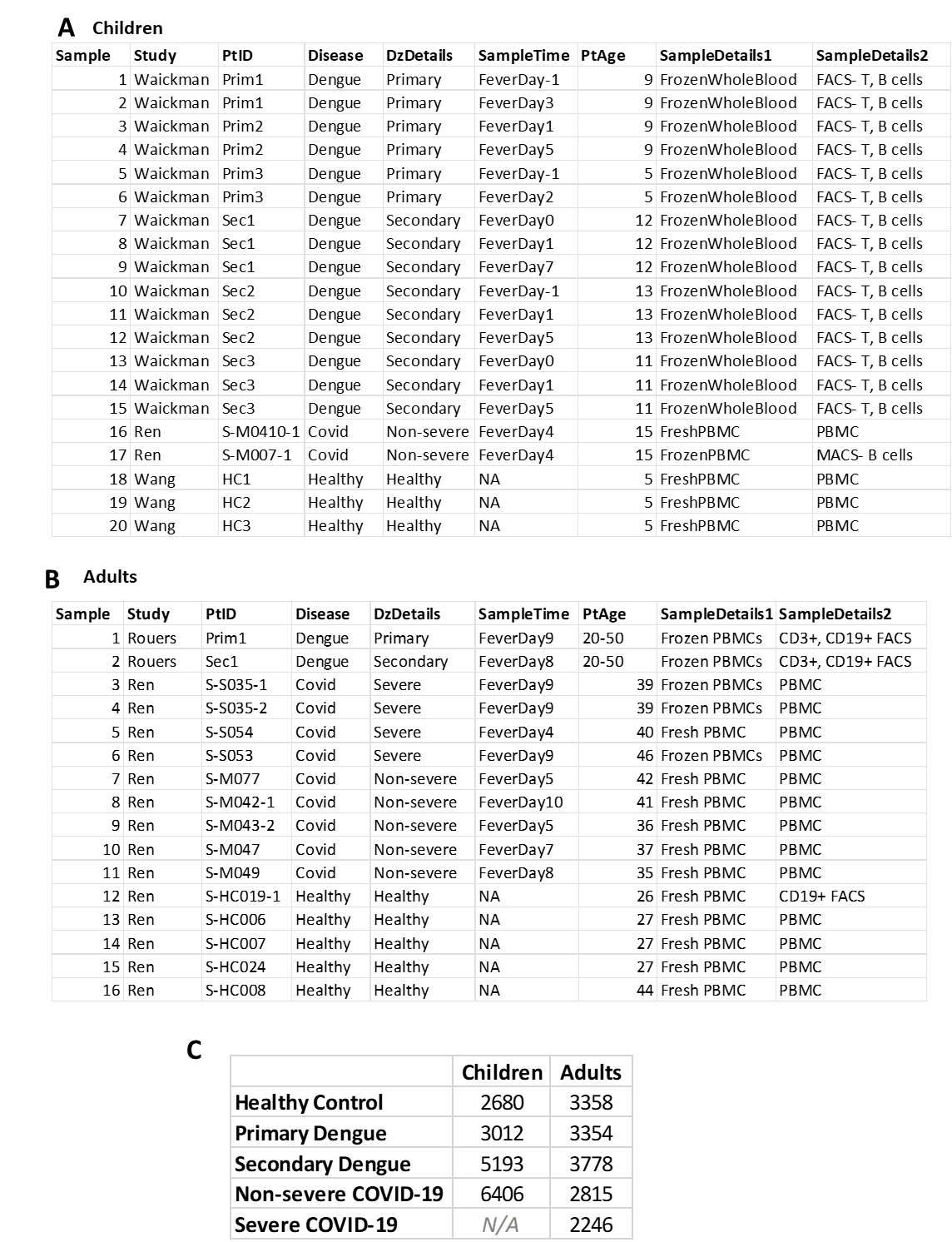


# Supplementary Figure 1

A. Meta-data for pediatric patients, with study of origin, patient ID, disease characteristics, sampling time with respect to symptom onset, patient age (in years), and specimen characteristics. FACS= fluorescence activated cell sorting, PBMC = peripheral blood mononuclear cells, MACS = magnetic activated cell sorting

B. Meta-data for adult patients, with study of origin, patient ID, disease characteristics, sampling time with respect to symptom onset, patient age (in years), and specimen characteristics.

C. Summary table of diseases and patients included in study with corresponding number of cells (after quality control) in each category; there are no pediatric patients in the severe COVID-19 category in this dataset.

#
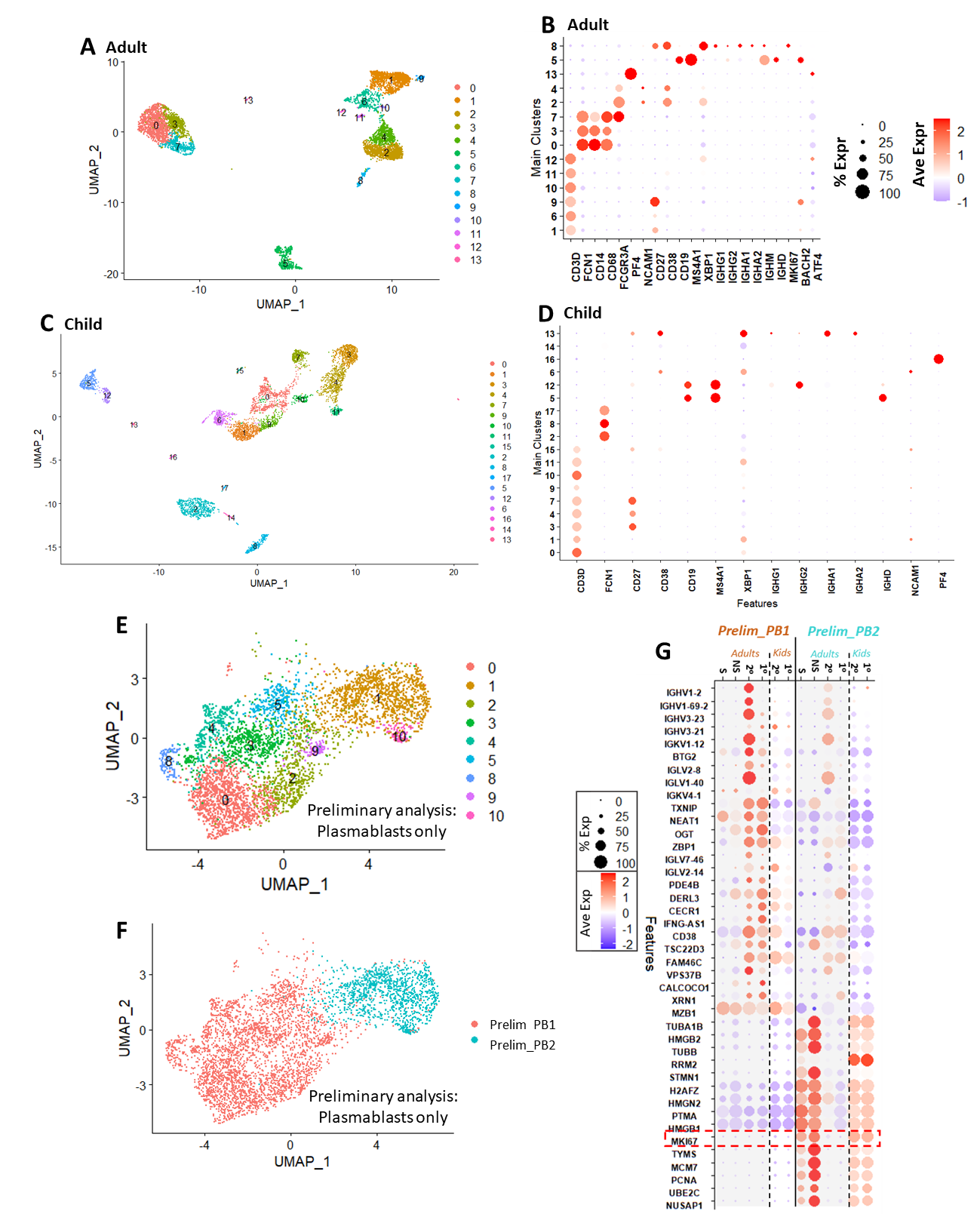


# Supplementary Figure 2

A. UMAP embedding for one representative adult patient, prior to integration with other adult patients for downstream analyses.

B. Cluster identification for UMAP in (A) using canonical immune cell markers: CD3 for T cells; FCN1, CD14, CD68 for monocytes; PF4 for platelets; NCAM1 for NK cells; CD19, MS4A1 for B cells; CD27, CD38, Ig heavy chains for plasmablasts

C. UMAP embedding for one representative pediatric patient, prior to integration with other pediatric patients for downstream analyses.

D. Cluster identification for UMAP in (B) using canonical immune cell markers.

E. Initial UMAP embedding of only plasmablast cells from children and adults as a preliminary analysis

F. UMAP embedding from (E) with initial clusters grouped into two cell subtypes: Prelim_PB1 (no MKI67 expression) and Prelim_PB2 (MKI67 expression).

G. Significant (p-adj <=0.05) cluster markers calculated with respect to groups Prelim_PB1 and Prelim_PB2, displayed with respect to disease condition and age. Red box shows MKI67 expression in adults with COVID-19 and children with dengue.

#
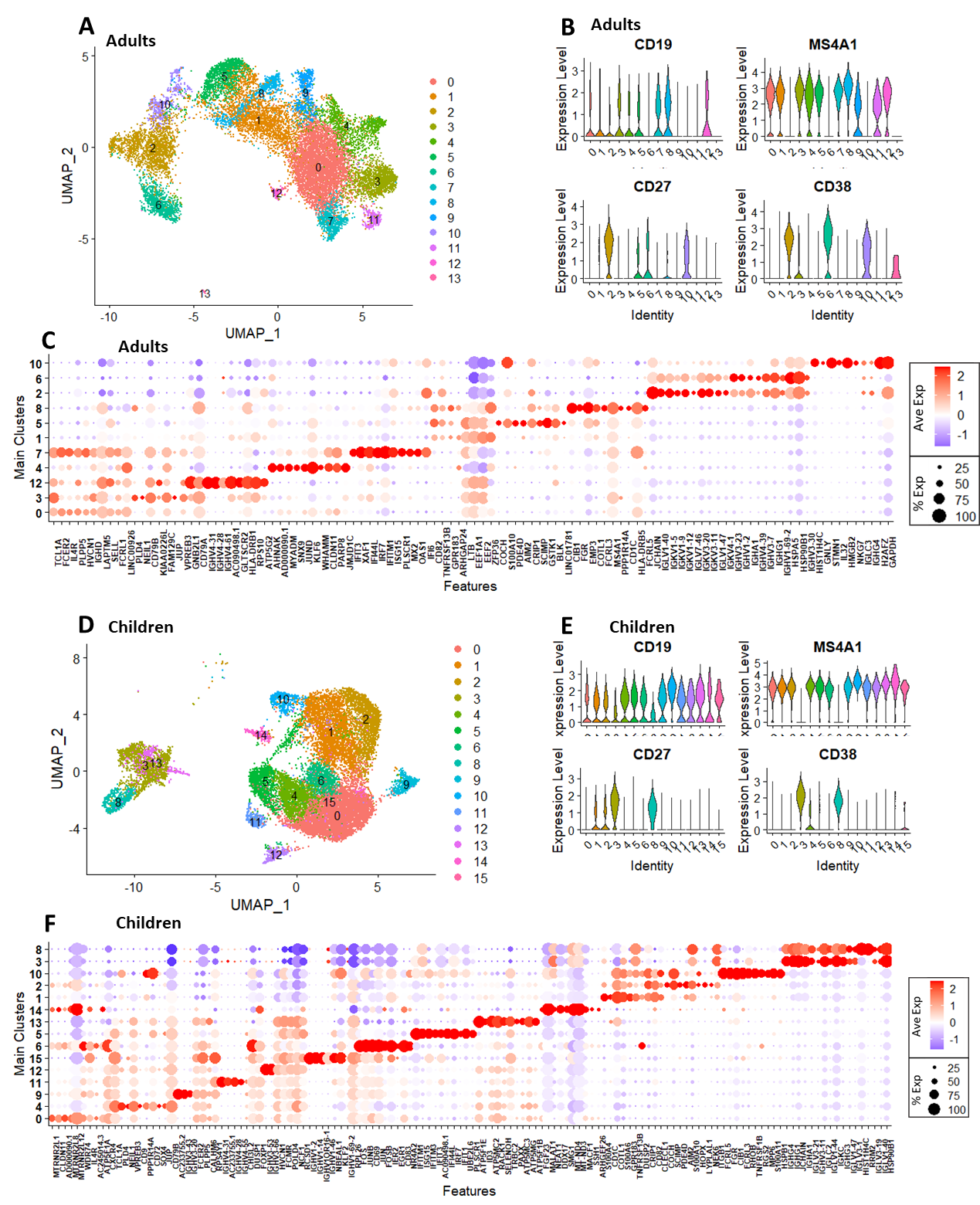


# Supplementary Figure 3

A. Initial UMAP embedding for all B cells from adult patients.

B. Violin plot of CD19, MS4A1; CD27, CD38 expression in initial clusters as determined in (A). Clusters retained as naive and memory B cells were CD19+/MS4A1+, while those retained as plasmablasts were CD27+/CD38+.

C. Top ten significant (p-adj <=0.05) cluster markers calculated with respect to initial clusters as determined in (A).

D. Initial UMAP embedding for all B cells from pediatric patients.

E. Violin plot of CD19, MS4A1; CD27, CD38 expression in initial clusters as determined in (D)

F. Top ten significant (p-adj <=0.05) cluster markers calculated with respect to initial clusters as determined in (D).

#
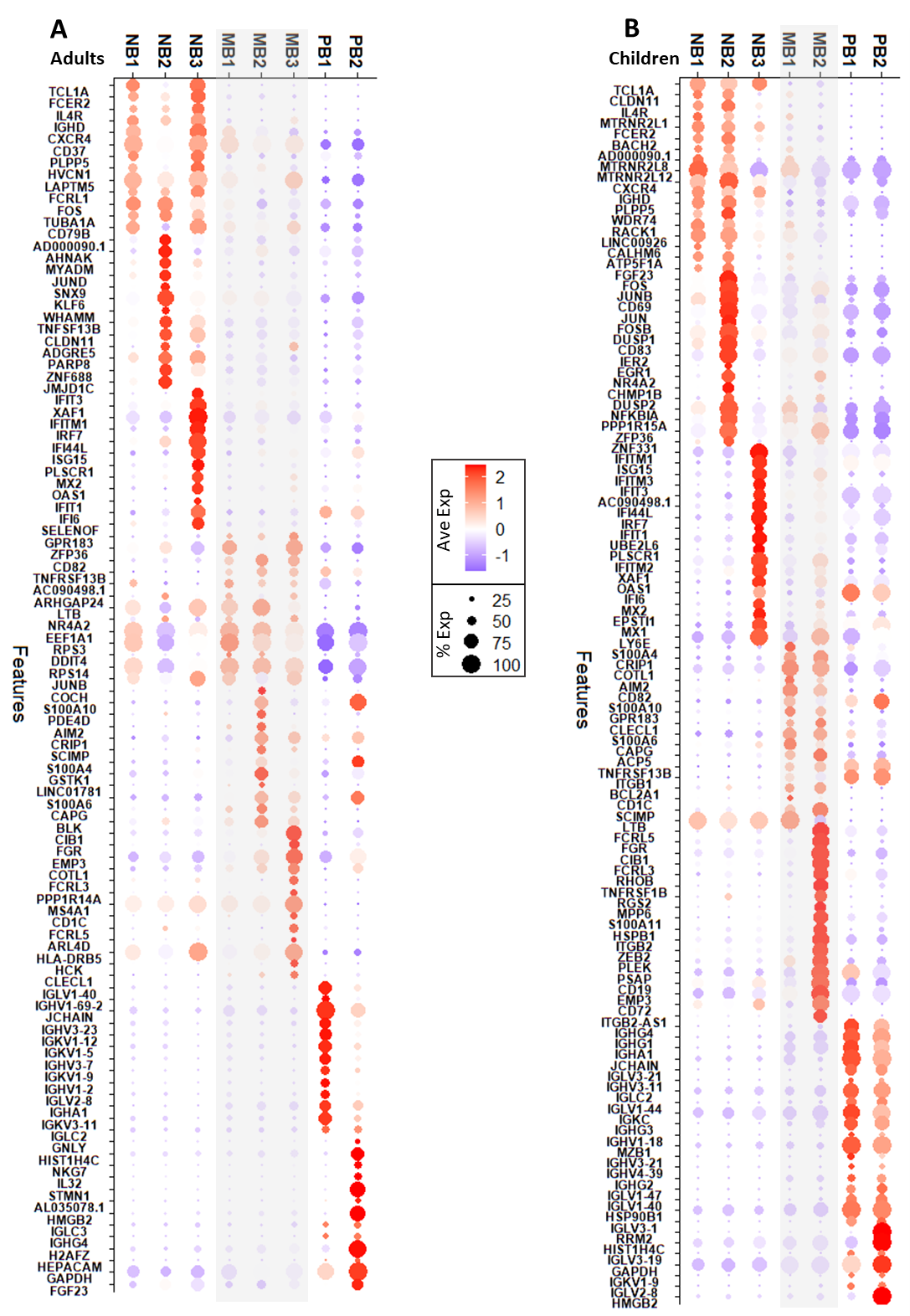


# Supplementary Figure 4

A. Top 15 significant (p-adj <=0.05) cluster markers calculated with respect to cell subtype in adult patients.

B. Top 15 significant (p-adj <=0.05) cluster markers calculated with respect to cell subtype in pediatric patients.

#
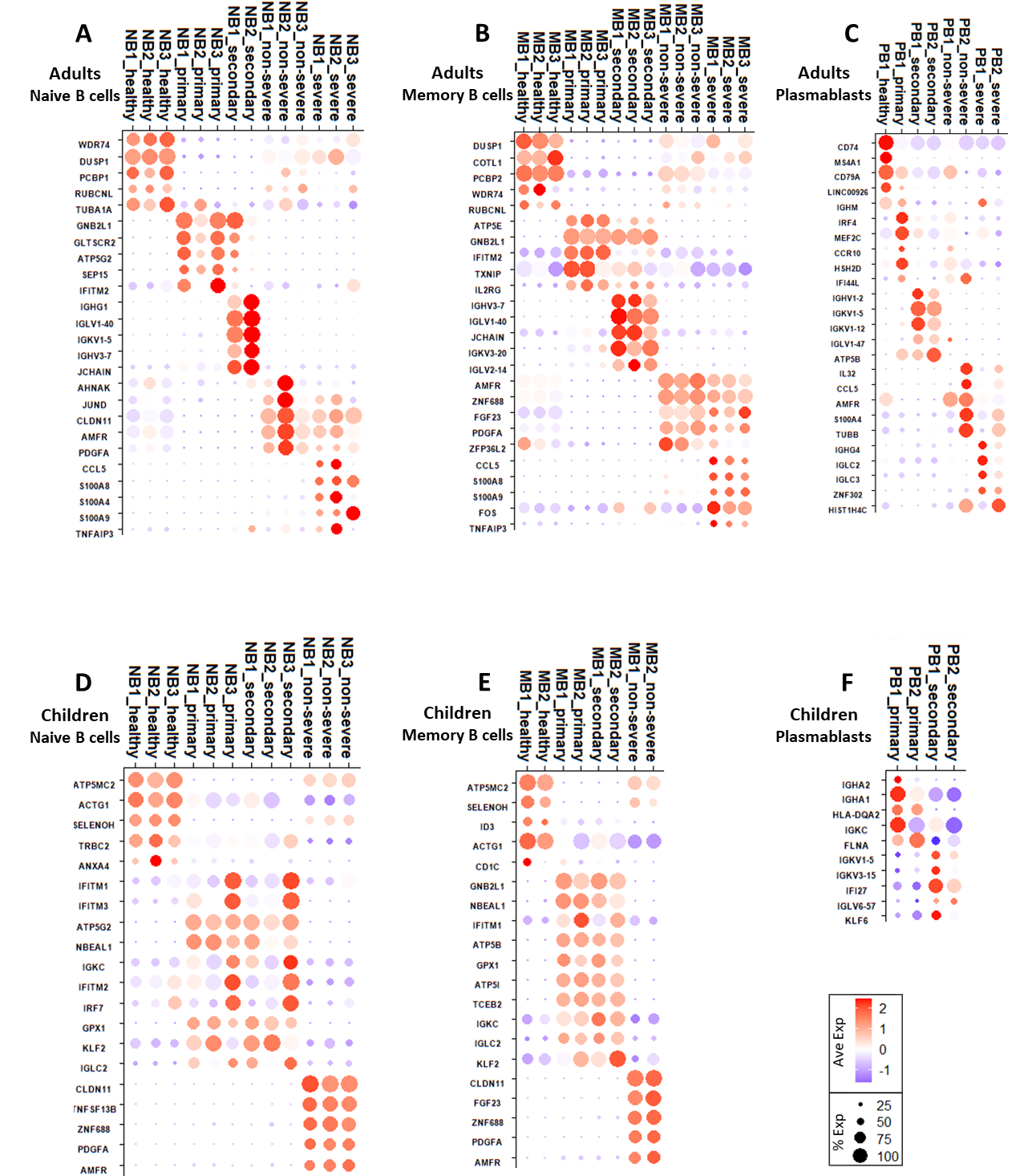


# Supplementary Figure 5

A-C. Top 5 significant (p-adj <=0.05) markers calculated with respect to disease condition (displayed with respect to disease condition and cell subtype) for naive B, memory B, and plasmablast cells, respectively, in adults.

D-F. Top 5 significant (p-adj <=0.05) markers calculated with respect to disease condition (displayed with respect to disease condition and cell subtype) for naive B, memory B, and plasmablast cells, respectively, in children.

#
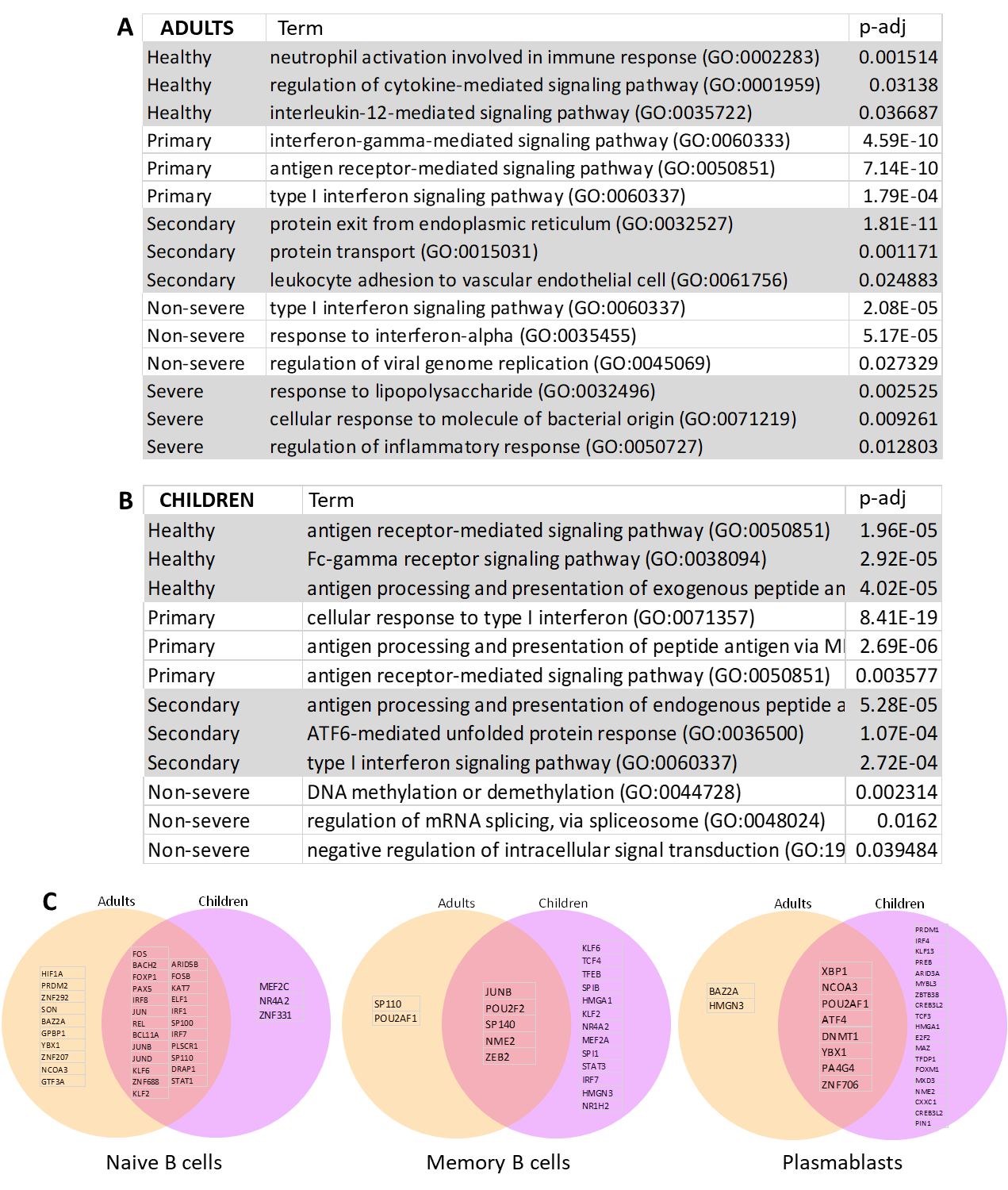


# Supplementary Figure 6

A,B. Gene ontology biological process (GOBP) terms calculated using significant (p-adj <=0.05) markers with respect to each disease condition (top 5 genes from each are shown in Supplemental Fig 5), for adults and children respectively.

C. Transcription factors (from significant cluster markers with respect to cell subtype and disease condition) that are unique and shared in adults and children across naive B, memory B, and plasmablast cells.


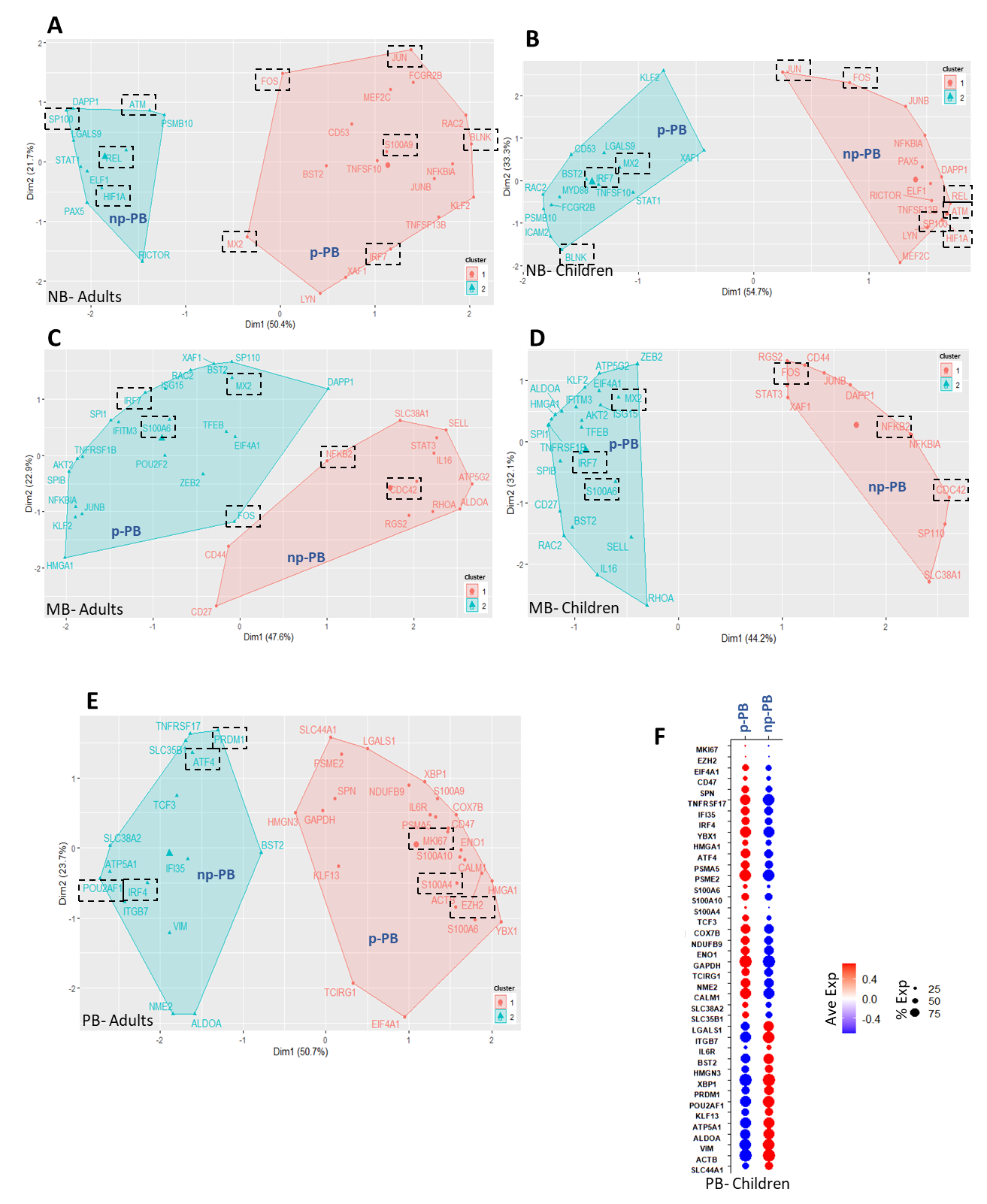


# Supplementary Figure 7

A, B. Cluster plot of result of k means clustering of genes with respect to average expression across disease conditions for naive B cells (NB) in adults and children, respectively. Boxes represent genes highlighted in network in figure 3. Genes with increased expression in groups either with or without p-PBs generally cluster together. Clusters that correspond to p-PB and np-PB are labeled.

C, D. Cluster plot of result of k means clustering of genes with respect to average expression across disease conditions for memory B cells (MB) in adults and children, respectively. Boxes represent genes highlighted in network in figure 4. Genes with increased expression in groups either with or without p-PBs generally cluster together. Clusters that correspond to p-PB and np-PB are labeled.

E. Cluster plot of result of k means clustering of genes with respect to average expression across disease conditions for plasmablast cells (PB) in adults. Boxes represent genes highlighted in network in figure 5. Genes with increased expression in groups either with or without p-PBs generally cluster together. Clusters that correspond to p-PB and np-PB are labeled.

F. Cluster plot of k means could not be generated since there are only two disease conditions (primary and secondary) for plasmablasts from children. Expression of genes highlighted in network in figure 5 is shown in the dotplot. Genes that correspond to p-PB and np-PB are labeled.


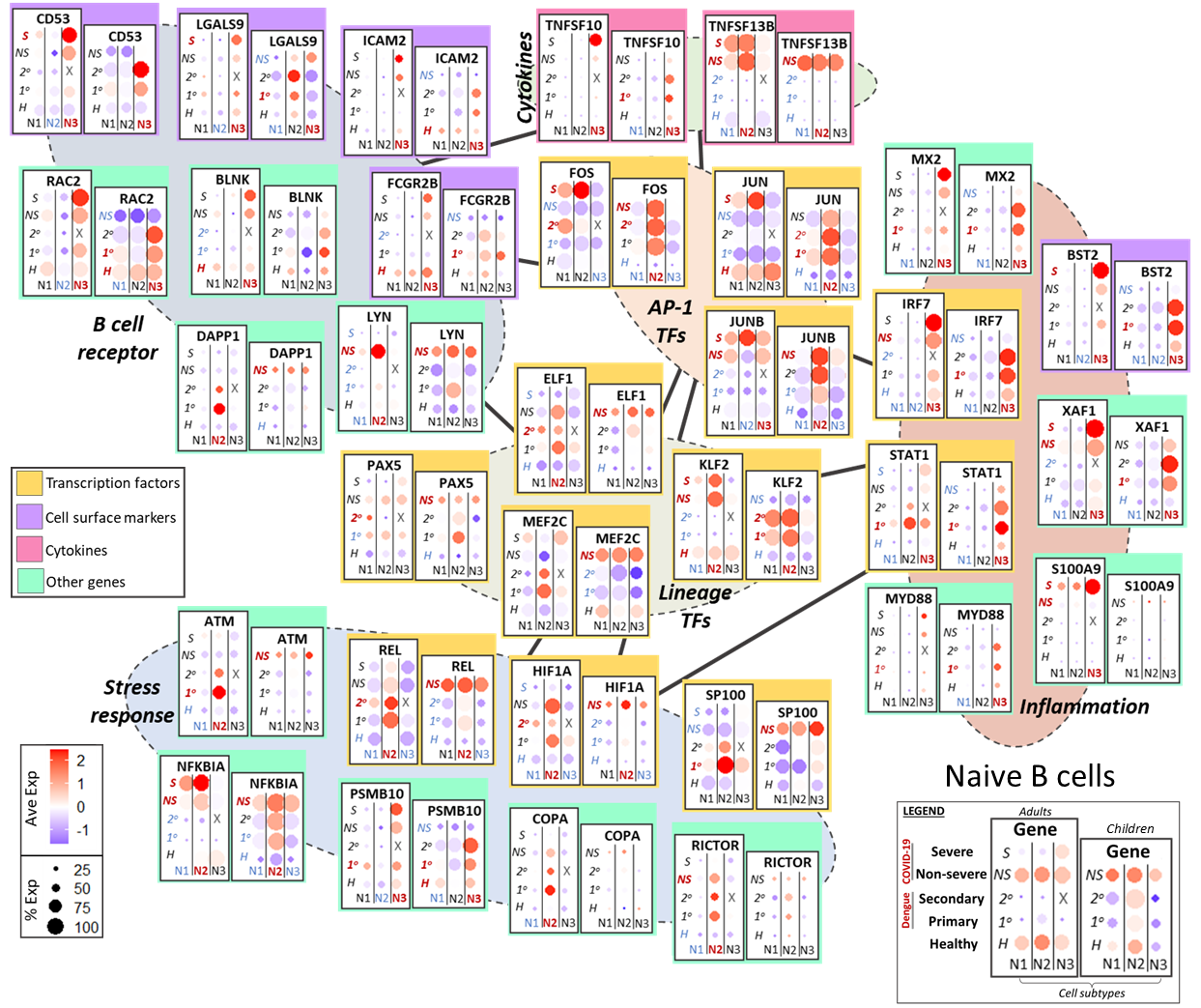


# Supplementary Figure 8

Expanded network of functional associations between key genes in naive B cell function and differentiation, including core transcription factors (TFs), interferon (IFN) response, B cell receptor components, cytokines, and cell surface molecules. See also Fig. 3.


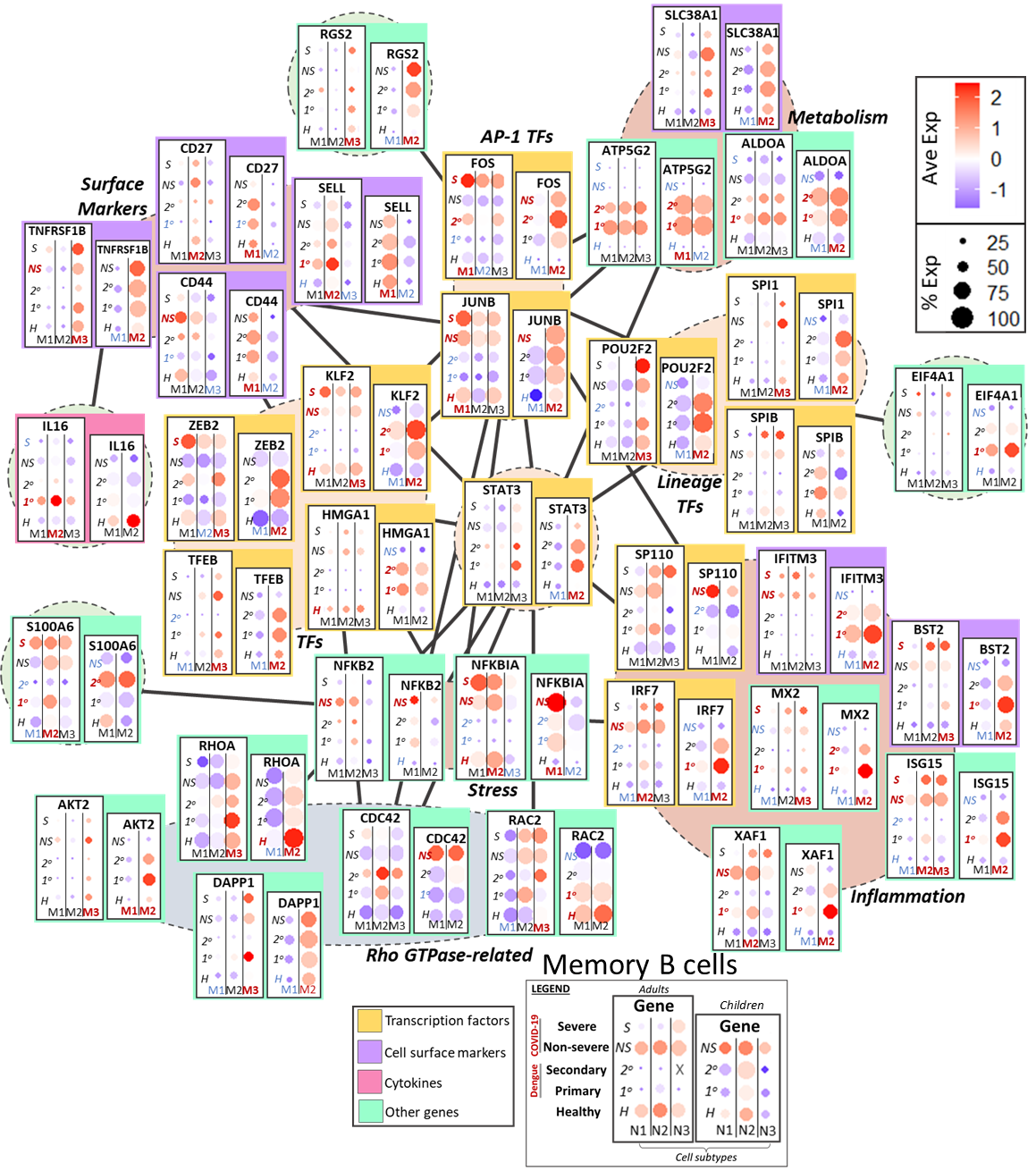


# Supplementary Figure 9

Expanded network of functional associations between key genes in memory B cell function and differentiation, including core transcription factors (TFs), interferon (IFN) response, B cell receptor components, cytokines, and cell surface molecules. See also Fig. 4.


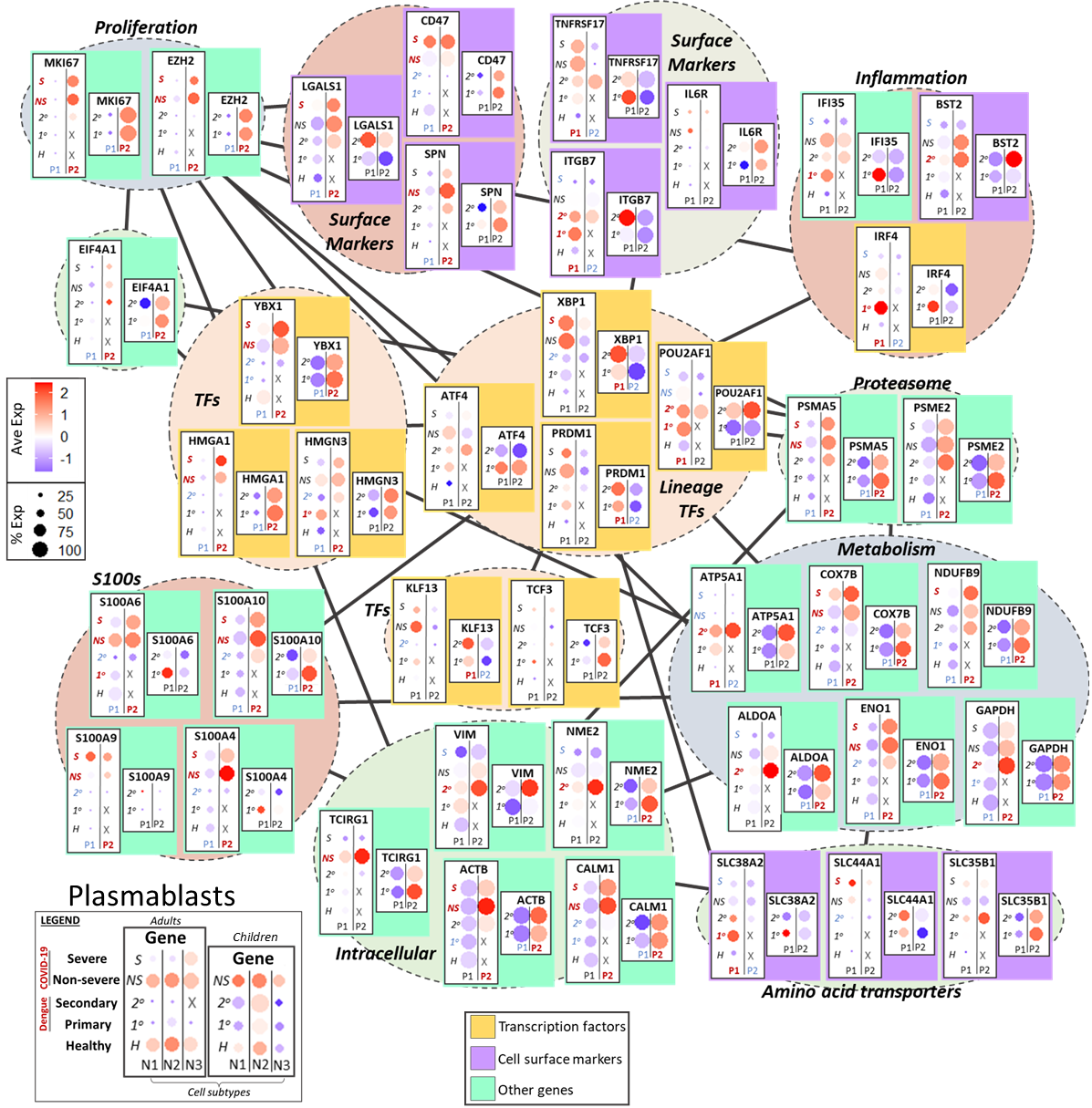


# Supplementary Figure 10

Expanded network of functional associations between key genes in plasmablast cell function and differentiation, including core transcription factors (TFs), interferon (IFN) response, B cell receptor components, and cell surface molecules. See also Fig. 5.


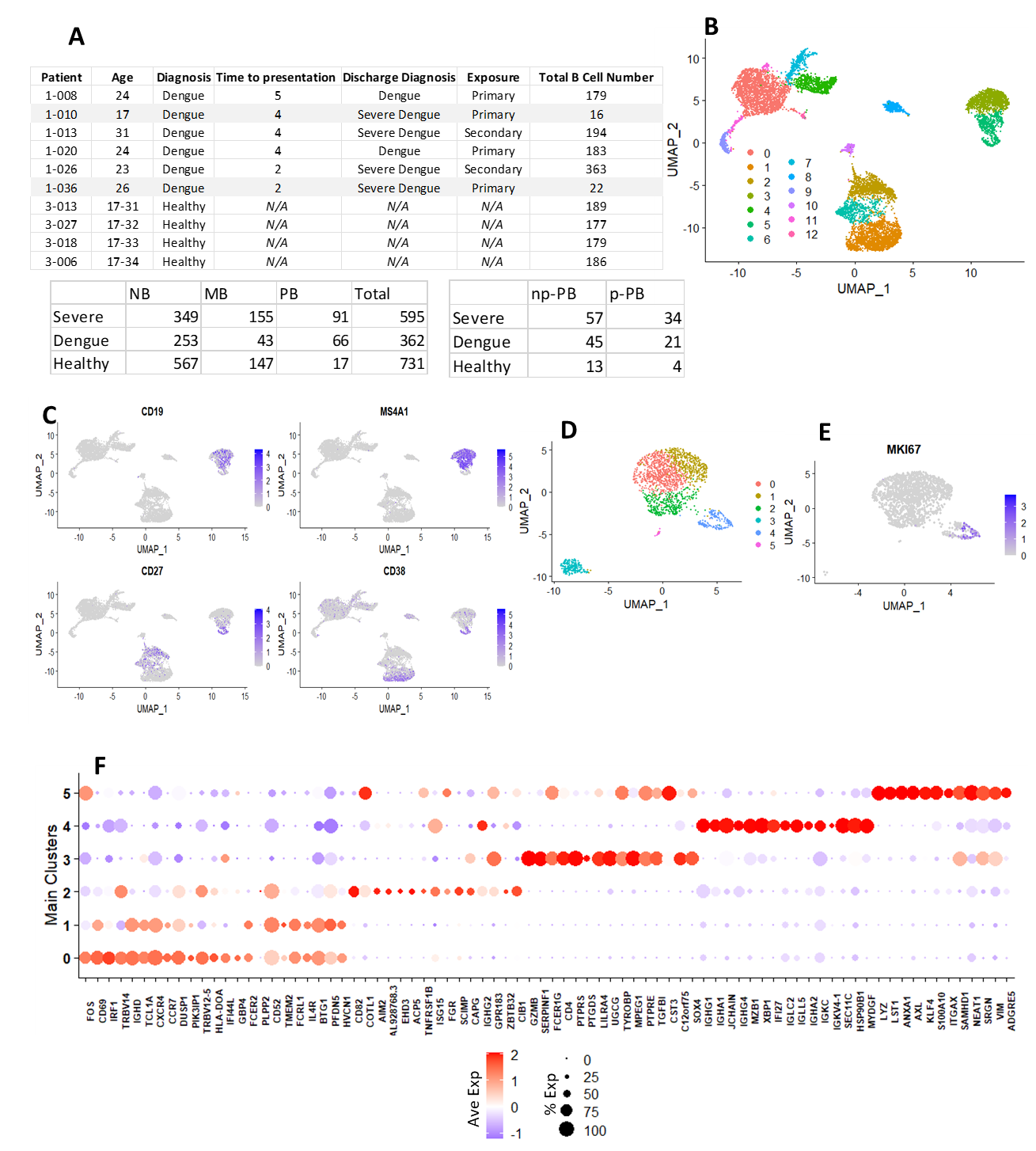


# Supplementary Figure 11

A. Metadata from patients from study of adult dengue patients by Zanini et al, which constituted an independent scRNA seq data set for validation of correlation of observed gene expression patterns in groups with and without p-PBs. Patients with low B cell count (grey) were excluded. The retained patients included 4 dengue patients (two with primary dengue, two with secondary) and four age-matched healthy controls. Raw B cell count after QC are shown below for each B cell type and disease condition. PB counts are further broken down into np-PB and p-PB cell counts for each disease condition.

B. UMAP representation of all PBMCs sequenced, after initial quality control and data integration.

C. Identification of B cell subsets with expression of CD19^+^/MS4A1^+^ representing naive and memory B cells, and CD27^+^/CD28^+^ representing plasmablasts.

D. UMAP representation of subset and reclustered B cells only

E. Expression of MKI67 in subset and reclustered B cells

F. Cluster markers (p <=0.05) calculated for clusters identified in part (D). These were used to determine which clusters represent naive B cells (clusters 0,1), memory B cells (cluster 2), and plasmblasts (cluster 4). Other clusters had markers more consistent with non-B cell types and were excluded from downstream analysis.
